# Predictors of loss to follow-up among children on long-term antiretroviral therapy in Zambia (2003–2015)

**DOI:** 10.1186/s12889-019-7374-0

**Published:** 2019-08-15

**Authors:** Jane N. Mutanga, Simon Mutembo, Amara E. Ezeamama, Xiao Song, Robert C. Fubisha, Kunda Mutesu-Kapembwa, Derrick Sialondwe, Brenda Simuchembu, Jelita Chinyonga, Philip E. Thuma, Christopher C. Whalen

**Affiliations:** 1 Department of Pediatrics and Child Health, Livingstone Central Hospital, Akapelwa Street, Livingstone, Zambia; 2 Department of Epidemiology and Biostatistics, College of Public Health, University of Georgia, Athens, GA USA; 3 Southern Province Medical Office, Ministry of Health, Choma, Zambia; 4 Department of Psychiatry, College of Osteopathic Medicine, Michigan State University, East Lansing, MI USA; 5 Macha Research Trust, Choma, Zambia

**Keywords:** Pediatrics, HIV, ART, Adherence, Loss to follow-up, Risk factors

## Abstract

**Background:**

Retention in care is critical for children living with HIV taking antiretroviral therapy (ART). Loss to follow-up (LTFU) is high in HIV treatment programs in resource limited settings. We estimated the cumulative incidence of LTFU and identified associated risk factors among children on ART at Livingstone Central Hospital (LCH), Zambia.

**Methods:**

Using a retrospective cohort study design, we abstracted data from medical records of children who received ART between 2003 and 2015. Loss to follow-up was defined as no clinical and pharmacy contact for at least 90 days after the child missed their last scheduled clinical visit. Non-parametric competing risks models were used to estimate the cumulative incidence of death, LTFU and transfer. Cause-specific Cox regression was used to estimate the hazard ratios of the risk factors of LTFU.

**Results:**

A total of 1039 children aged 0–15 years commenced ART at LCH between 2003 and 2015. Median duration of follow-up was 3.8 years (95% CI: 1.2–6.5), median age at ART initiation was 3.6 years (IQR: 1.3–8.6), 179 (17%) started treatment during their first year of life. At least 167 (16%) were LTFU and we traced 151 (90%). Of those we traced, 39 (26%) had died, 71 (47%) defaulted, 20 (13%) continued ART at other clinics and 21 (14%) continued treatment with gaps. The cumulative incidence of LTFU for the entire cohort was 2.7% (95% CI: 1.9–3.9) at 3 months, 4.1% (95% CI: 2.9–5.4) at 6 months and 14.1% (95% CI: 12.4–16.9) after 5 years on ART. Associated risk factors were: 1) non-disclosure of HIV status at baseline, aHR = 1.9 (1.2–2.9), 2) No phone ownership, aHR = 2.1 (1.6–2.9), 3) starting treatment between 2013 to 2015, aHR = 5.6 (2.2–14.1).

**Conclusion:**

Among the children LTFU mortality and default were substantially high. Children who started treatment in recent years (2013–2015) had the highest hazard of LTFU. Lack of access to a phone and non-disclosure of HIV-status to the index child was associated with higher hazards of LTFU. We recommend re-enforcement of client counselling and focused follow-up strategies using modern technology such as mobile phones as adjunct to current approaches.

## Background

Retention in care and viral suppression are optimal outcomes for children living with HIV taking lifelong Antiretroviral Therapy (ART) [1]. Good adherence to medication is critical for viral suppression. Poor medication adherence results in development of HIV viral resistance and subsequently treatment failure. Treatment failure is undesirable because ART is lifelong and there are few drug choices especially in resource limited settings. To achieve optimum adherence and viral suppression, retention in care is critical in ART programs.

Retention in care is particularly challenging in pediatric HIV treatment programs in Sub-Sahara Africa where the proportion of children lost to follow-up (LTFU) has been estimated to be around 9–14% during the first year of treatment and up to 28% during the second year of treatment [2]. Disruption in HIV care because of missed appointments can undermine clinical outcomes including assessment of adverse events, ongoing provision of prophylactic medications, clinical and neurodevelopment assessment and early identification of treatment failure [3].

Most studies of LTFU in Sub-Sahara Africa focus on shorter periods of follow-up time and the findings vary widely across different settings [4–9]. We carried out a retrospective analysis of children living with HIV taking ART and estimated the cumulative incidence of LTFU and identified associated risk factors among children on ART at Livingstone Central Hospital (LCH) in Zambia.

## Methods

### Study site and population

We conducted a retrospective cohort study of infants and children who received ART at the Pediatric Center of Excellence clinic (PCOE), an outpatient children’s clinic at LCH in Southern Province, Zambia. Livingstone Central Hospital serves over 200,000 people in the Southern Province and parts of the Western Province of Zambia. Pediatric HIV treatment at LCH was started in 2003 in line with national policy and in 2006 the PCOE clinic was established through a collaborative agreement between the Ministry of Health in Zambia and the Centers for Diseases Control and prevention country office (CDC) [10].

We analyzed data for children with confirmed HIV infection and receiving ART at the PCOE between January 2003 and December 2015. The inclusion criteria were: 1) children aged from birth to 15 years at the time of ART initiation who commenced ART between January 1st 2003 and December 31st 2015, 2) children who had taken ART for at least 6 months or more, 3) children who had received at least three ARVs. Children who received one ARV for PMTCT were excluded from the study because we considered ART to be a combination of at least three antiretroviral drugs. We excluded children who started ART on or after June 30th, 2015 because data collection was closed on December 31st, 2015 and we included only children who took ART for at least 6 months.

The date of entry into the cohort was the date the child began taking ARVs as recorded on the medical chart. Children who were transferred out of the clinic and children who died were censored on the date the outcomes were ascertained. All the children who were lost to follow-up were censored at least 90 days after they missed their last clinic visit. Children who were active in care were censored on December 31st, 2015 when the study was closed. Adolescents who transitioned to adult care were censored on the date of their last visit and were recorded as transferred out of the clinic.

### Study outcome and exposure

The main outcome was LTFU which was defined as no clinical and pharmacy contact for at least 90 days after the child missed their last scheduled clinical visit. The exposure was ART. Since this was a retrospective cohort study, the decision to initiate ART was made by the treating physician and the child’s caregivers and not influenced by the study investigators.

### Clinical management

Clinical management of the study population was based on the Ministry of Health in Zambia pediatric HIV treatment guidelines. The 2007 pediatric HIV testing and treatment guidelines recommended virologic testing where available and presumptive HIV diagnosis and treatment of HIV exposed infants and children under 18 months of age who presented with WHO stage 3 and 4 conditions or had low CD4% [11]. The 2013 pediatric HIV testing and treatment guidelines recommended collection of dried blood spots (DBS) cards for DNA-PCR from all HIV-exposed infants at 6 weeks and 6 months of age followed by serologic testing at 12 months, 18 months and 3 months after cessation of breastfeeding [12]. Routine provider initiated counseling and testing was recommended for all hospitalized children. All children who were confirmed HIV seropositive were initiated on ART. Prior to 2012, pediatric HIV treatment guidelines recommended ART based on clinical and immunological criteria. First line ART regimens comprised of three antiretroviral medicines including two Nucleoside reverse transcriptase inhibitors (NRTI’s): Azidothymidine, Stavudine, Abacavir, (Tenofovir for children above 10 years old) and Lamivudine, plus one Non-Nucleoside Reverse Transcriptase Inhibitor (NNRTI), either Nevirapine or Efavirenz for ART naïve infants and children. Infants who took ART prophylaxis after delivery were commenced on Protease inhibitors (PI) based regimens (Lopinavir boosted with Ritonavir) in addition to two NRTIs. Triple NRTI based regimens were recommended for children below 3 months of age who were co-infected with Tuberculosis at baseline [12].

Children who tested positive for HIV were enrolled into care by trained nurse counsellors at the PCOE clinic using standardized forms. The data collected included; sex, date of birth, birth history, mother’s gestational history including date of HIV test and whether the mother took ARVs. The child’s caregivers contact information (phone numbers and home address, directions on how to get to the house using landmarks) were collected. Oral and written consent to contact the family in case they missed appointments was obtained and documented in the medical record. This information was updated by the registry clerks at every clinic visit. Once the patient was commenced on ART, they were scheduled to come back to the clinic after 2 weeks. Following the 2 weeks’ clinical visit, the patient was scheduled for monthly clinical visits for 6 months after which they could be scheduled for 3 monthly clinical visits if they were clinically stable and adherence was observed to be satisfactory. During the counseling process, the caregivers were informed that they could bring the child back for sick visits without a prior appointment. At each visit, the child’s weight, height, head circumference was measured and recorded in the medical chart. Developmental milestones were monitored and recorded in the medical charts for young children.

### Patient tracing procedures

The PCOE had an outreach team comprised of nurse counsellors, social worker and community volunteers. The clinic nurses kept a list of patients who did not come for their scheduled appointments every day and contacted those whom they could call by phone. At the end of the week, the outreach team collected the list of patients with missed appointments and tried to contact them either by phone or home visits. Attempts to contact the caregivers and re-engage the children were made over a period of 3 months. If the outreach team was not able to trace the child within a period of 3 months, the tracing outcome was classified as “LFTU” and the child’s medical chart was flagged and kept in a separate registry folder. Tracing was successful when the outreach team found a child and determined their outcome.

For this study, we collected all the medical charts in the “LFTU” registry folder and attempted to trace the children. When a child was traced and their caregivers decided not to continue with medical treatment despite the interventions, the child’s tracing outcome was classified as “Defaulter”. When a child was found, and they had continued taking ART at another facility the tracing outcome was “on ART at other facility”. If the child had a period when they were not taking ART but came back to the clinic and continued treatment, the tracing outcome was “on ART with gaps”. And if the child was traced but had died, the tracing outcome was “dead”. Children who were not found were classified as “unknown status”.

For all the children who were traced with outcomes “dead”, “defaulted”, “On ART at other facility”, “on ART with treatment gaps” and “unknown status” the exact date the outcome occurred was not available and we used the date the outcome was ascertained by the outreach team as documented on the medical records.

We examined the medical records of all the patients at the clinic and compared with the outreach team’s registers to ensure that we did not miss any additional patients who were lost to follow-up. We looked for patients who had been lost to follow-up but silently returned to care by comparing the medical records with the outreach team’s registers and the clinic registers.

### Data collection

Patient data were abstracted from the medical records of children who received ART. We created a Microsoft Access database with the following variables; date of birth, gender, date of ART initiation, weight, height, WHO stage, CD4 count, hemoglobin, viral load, mother’s PMTCT history, delivery history, medical history, ART regimens, adherence history, social economic indicators of the care giver including occupation, income, phone ownership, visit dates and treatment outcome. We assigned unique study identification numbers to each patient record. Data abstraction was done independently by two data entry clerks and their entries were verified at random intervals by a supervisor.

### Data analysis

Baseline demographics and clinical features were described by estimating medians and interquartile ranges for continuous variables and frequencies and proportions for categorical variables. Non-parametric competing risks models were used to estimate the incidence of death, transfer and LTFU [13]. We estimated the cumulative incidence at 3 months, 6 months, 1 year, 3 years, 5 years and 10 years of observation in the competing risks model. Our event of interest in the analysis was the initial LTFU because most of the patients had been lost for more than 90 days before we traced them for the purpose of our study. Cause-specific Cox Proportional Hazards regression was used to estimate the hazard ratios of the risk factors of loss to follow-up. All statistically significant variables (*p* < 0.05) in the univariable analysis were included in the multivariable analysis. We evaluated the proportional hazards assumption using log-log plots and plots of Schoenfeld’s residuals and no violations of the assumption were found. The dataset was almost complete as we had very few missing variables.

Data analysis was done using R statistical software version 3.4 [14]. We used the cmprsk package for the competing risks analysis [15].

## Results

**Fig. 1:**
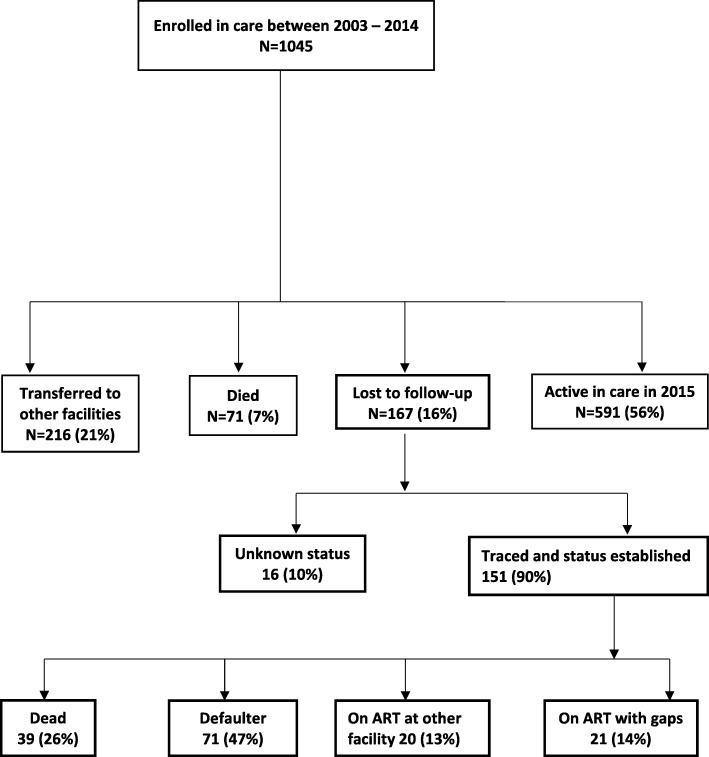
Treatment outcomes and retention in care among children on ART at LCH (2003–2014)

A total of 1039 children aged less than 15 years old commenced ART at LCH between January 2003 and June 2015. At the time of our analysis, 591 (56%) children were alive and active in care, 210 (20%) had transferred to other facilities, 71 (7%) had died and 167 (16%) were lost to follow-up. Of the 167 who were lost to follow-up, we traced 151 (90%) and did not find 16 (10%). Of the 151 children that we found, 39 (26%) had died, 71 (47%) had stopped treatment (defaulters), 20 (13%) continued ART at other clinics and 21 (14%) had continued treatment with gaps (Fig. 1).

### Baseline characteristics of children on ART

The median duration of follow-up was 3.8 years (95% CI: 1.2–6.5) and the median age at baseline was 3.6 years (IQR: 1.3–8.6). At the time of ART initiation, 520 (49%) children were female and 721 (69%) were cared for by their biological mothers. A total of 179 (17%) commenced treatment during their first year of life. In total, 49% (512) of children commenced ART between 2006 and 2009 but this declined to 29% (305) of children between 2010 and 2012 and 14% (151) between (2013–2015). At least 304 (29%) were diagnosed during hospital admission and 30 (3%) were diagnosed at the time of birth, while their mothers were still admitted to the delivery wards (Table 1). At least 68% of the caregivers owned phones. HIV status was only disclosed to children aged 7 years old and above as recommended by national guidelines and HIV disclosure had been completed for 18% (*n* = 191) of children at the time of ART initiation (Table 1).

**Table 1:** Baseline Characteristics for Children commencing ART at LCH (2003–2015)

Characteristic	< 1 year	1–5 years	6–15 years	Total
	*N* = 179 (17%)	*N* = 387 (37%)	473 (46%)	*N* = 1039
Gender n(%)				
Female	84 (16%)	182 (35%)	254 (49%)	520
Male	95 (18%)	205 (40%)	219 (42%)	519
Age (years) at ART initiation Median (IQR)	0.5 (0.3–0.8)	2.0 (1.4–3.2)	10.2 (7.4–13)	3.6 (1.3–8.6)
Duration (years) of observation Median (IQR)	2.1 (0.5–5.2)	4.2 (1.2–6.5)	4.1 (1.9–6.7)	3.8 (1.2–6.5)
Duration of time (weeks) from diagnosis to ART initiation Median (IQR)	7 (2–11)	5 (2–15)	7 (2–39)	6 (2–19)
Who is the child’s Guardian				
Mother	150 (21%)	316 (44%)	255 (35%)	721
Father	5 (11%)	8 (18%)	30 (68%)	44
Grandparent	6 (8%)	24 (32%)	46 (61%)	76
Sibling	0	1 (6%)	14 (88%)	16
other relative	5 (4%)	22 (17%)	99 (79%)	126
*missing*	*13 (22%)*	*16 (28%)*	*29 (50%)*	*56*
Point of Entry into HIV care				
Out-patients departments	22 (13%)	44 (25%)	110 (63%)	176
Inpatient Wards	80 (26%)	152 (50%)	72 (24%)	304
MCH/Delivery wards	5 (17%)	17 (57%)	8 (27%)	30
VCT clinic (FSU)	11 (9%)	42 (33%)	72 (57%)	127
TB clinic	3 (25%)	3 (25%)	6 (50%)	12
*missing*	*58 (15%)*	*129 (33%)*	*205 (52%)*	*390*
Educational level of caregiver				
None	2 (10%)	11 (58%)	6 (52%)	19
Primary or secondary school	113 (18%)	226 (37%)	272 (44%)	612
Some college or university	11 (18%)	16 (27%)	32 (53%)	60
*missing*	*53 (15%)*	*134 (38%)*	*163 (47%)*	*348*
Does the family have a phone				
Yes	102 (14%)	243 (35%)	358 (51%)	705
Has HIV status been disclosed to the child				
Yes	0	0	191 (100%)	191

The median baseline CD4+ count for children 6 to 15 years old was 505 (IQR: 243–948). Whereas for children between 1 and 5 years the median CD4% was 19.4% (IQR: 12.4–25.6), children aged less than 1 year had median CD4% of 16.7% (IQR: 11.3–21.5). At least 1002 (97%) took Cotrimoxazole at baseline and 301 (30%) had a diagnosis of clinical Tuberculosis (TB) at ART initiation. Overall, 472 (46%) were WHO stage 3 and 177 (17%) were WHO stage 4 (Table 2).

**Table 2:** Baseline Laboratory and Clinical characteristics of Children commencing ART at LCH

Characteristic	< 1 year	1–5 years	6–15 years	Total
	*N* = 179 (17%)	*N* = 387 (37%)	*N* = 473 (46%)	*N* = 1039
CD4 count at enrollment Median (IQR)	**1028 (535–1498)**	**777 (444–1135)**	**278 (118–487)**	**505 (243–948)**
CD4 percent at enrollment Median (IQR)	**19.4 (12.4–25.6)**	**16.7 (11.3–21.5)**	**14.0 (8.3–21.2)**	16.2 (10–23)
Hemoglobin at enrollment (Median IQR))	**8.8 (7.8–9.7)**	**9.3 (8.0–10.5)**	**10.3 (9–11.5)**	**9.6 (8.3–10.9)**
Taking Cotrimoxazole at enrollment				
Yes	169 (17%)	378 (38%)	451 (45%)	1000
No	9 (23%)	8 (21%)	22 (56%)	39
Drug regimen at ART initiation N(%)				
3 NRTI’s	30 (38%)	46 (59%)	2 (3%)	78
2NRTIs +1NNRTI	130 (14%)	320 (35%)	457 (50%)	907
LPV/r based	19 (35%)	21 (39%)	14 (26%)	54
Year of ART start N(%)				
2003–2005	3 (4%)	17 (24%)	51 (72%)	71
2006–2009	88 (17%)	202 (39%)	222 (43%)	512
2010–2012	62 (20%)	117 (38%)	126 (41%)	305
2013–2015	26 (17%)	51 (34%)	74 (49%)	151
Mom took ARVs for PMTCT during pregnancy				
Yes	69 (45%)	71 (46%)	14 (9%)	154
No	110 (12%)	316 (36%)	459 (52%)	885
Child took ARV prophylaxis after birth N(%)				
Yes	55 (43%)	58 (47%)	8 (10%)	123
No	124 (14%)	329 (36%)	463 (51%)	810
Nutritional status at enrollment				
Weight for height score < −3SD	36 (20%)	88 (48%)	58 (32%)	182
weight for height score > −3 SD	143 (17%)	299 (35%)	415 (48%)	857
Baseline clinical staging (WHO stage) N(%)				
WHO stage 1	52 (24%)	64 (29%)	103 (47%)	219
WHO stage 2	20 (13%)	39 (25%)	99 (63%)	158
WHO stage 3	65 (14%)	200 (43%)	206 (44%)	471
WHO stage 4	41 (23%)	78 (44%)	58 (33%)	177
missing	1	6	7	14
Diseases at baseline N(%)				
TB	33 (10%)	105 (33%)	178 (56%)	318
pneumonia	35 (21%)	68 (41%)	61 (27%)	165
Diarrhea	45 (18%)	117 (47%)	84 (34%)	247
Gestation age at birth N(%)				
Premature	4 (25%)	8 (50%)	4 (25%)	16
term	107 (22%)	201 (41%)	175 (36%)	485
unknown	68 (13%)	178 (33%)	294 (54%)	544
Mode of delivery N(%)				
C/Section	5 (28%)	3 (17%)	10 (56%)	18
SVD	105 (21%)	206 (42%)	180 (37%)	491
unknown	69 (13%)	178 (34%)	283 (53%)	530

The first line regimen for 907 (87%) children was two NRTIs and one NNRTI, and 54 (5%) were commenced on two NRTIs and a Protease Inhibitor (Lopinavir boosted with Ritonavir) while 78 (8%) children took triple NRTIs at baseline, (Azidothymidine or Stavudine, Lamivudine and Abacavir) (Table 2).

Among LTFU children, 13% continued treatment with gaps and reported that they did not take pills for at least 2 weeks. The reported reasons for not taking pills included; distance from the health facilities, change of caregiver, unexpected travel for a prolonged period and the caregiver was busy with other activities. At least 13% of children were found to have continued treatment at another facility without informing the health facility staff. The reported reasons for the silent transfer were similar to those reported for treatment with gaps.

### Loss to follow-up

**Fig. 2:**
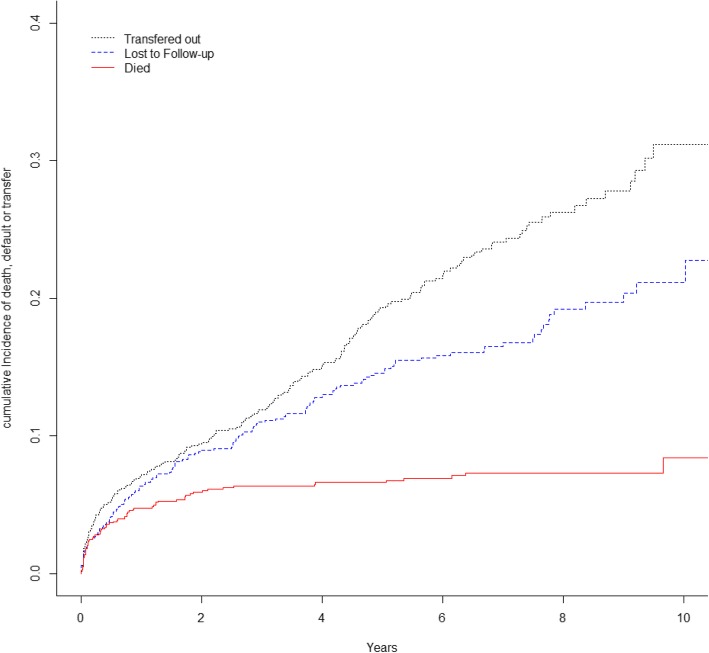
Cumulative Incidence Curve for children who died, transferred to another facility or were lost to follow-up based on a competing Risk Model

In the competing risks analysis, the cumulative incidence of LFTU for the entire cohort was 2.7% (95% CI: 1.9–3.9) at 3 months, 4.1% (95% CI: 2.9–5.4) at 6 months and continued to increase over time to 14.1% (95% CI: 12.4–16.9) and 21.1% (95% CI: 17.6–24.9) after 5 and 10 years on ART respectively (Fig. 2 and Table 3).

**Table 3:** Estimated Pointwise Cumulative Incidence of Lost to Follow-up, Transfer, and Death Based on Competing Risks Model Among Children on ART at LCH (2003–2015)

	3 months Cumulative incidence (95% CI)	6 months Cumulative incidence (95% CI)	1 year Cumulative incidence (95% CI)	3 years Cumulative incidence (95% CI)	5 years Cumulative incidence % (95% CI)	10 years Cumulative incidence % (95% CI)
Dead (entire cohort)	2.7 (1.9–3.9)	3.7 (2.6–4.9)	4.8 (3.6–6.2)	6.3 (4.9–7.9)	6.6 (5.2–8.3)	8.4 (5.9–11.3)
Transfer Out (entire cohort)	4.3 (3.1–5.6)	5.3 (4.1–6.8)	7.1 (5.6–8.7)	11.9 (9.9–14.0)	19.3 (16.6–22.0)	31.1 (26.5–35.9)
Lost to follow-up (entire cohort)	2.7 (1.9–3.9)	4.1 (2.9–5.4)	6.3 (4.9–7.9)	11.0 (9.2–13.1)	14.6 (12.4–16.9)	21.1 (17.6–24.9)

Loss to follow-up differed significantly across key baseline demographic, clinical and treatment variables. Cumulative incidence of LTFU increased over time for all age groups and was consistently higher among children who started ART at < 1 year of age: after 3 months of treatment cumulative incidence was 5% (95% CI: 2.5–8.9) for < 1 year, 3.6% (95% CI: 2.0–5.8) for children 1–5 years and 1.3% (95% CI: 0.5–2.6) for children 6–15 years, the *p*-value for difference across age strata was statistically significant (*p* < 0.0001) (Fig. 2, Table 4). Likewise, cumulative incidence of LTFU was higher among children acutely malnourished at baseline compared to children who were not acutely malnourished 5.0% (95% CI:2.4–8.8) after 3 months, (*p* = 0.0107). Children who commenced triple NRTIs had higher cumulative incidence of LTFU [10.3% (95% CI: 4.7–18.3)] after 3 months compared to those who were on standard first line ARV regimens (1NNRTI + 2NRTI) [2.2% (95% CI: 1.3–3.3)], and protease inhibitor based regimens [1.8% (95% CI: 0.1–8.7)] (*p* < 0.0001).

**Table 4:** Stratified Estimated Pointwise Cumulative Incidence of Loss to follow-up

	3 months Cumulative incidence % (95% CI)	6 months Cumulative incidence % (95% CI)	1 year Cumulative incidence % (95% CI)	3 years Cumulative incidence % (95% CI)	5 years Cumulative incidence % (95% CI)	10 years Cumulative incidence % (95% CI)	*p*-value
Age at ART Initiation^1^							< 0.0001
< 1 year	5.0(2.5–8.9)	9.0(5.3–13.7)	14.7(9.9–2.0)	22.3(16.3–28.8)	25.9(19.4–32.8)	27.7(20.9–34.9)	
1–5 years	3.6(2.0–5.8)	4.9(3.1–7.4)	7.6(5.2–10.5)	12.7(9.5–16.3)	14.8(11.3–18.8)	17.2(12.9–21.9)	
6–15 years	1.3(0.5–2.6)	1.5(0.7–2.9)	2.1(1.1–3.8)	5.3(0.3.5–7.7)	10.0(7.3–13.3)	20.3(14.8–26.2)	
Weight for Height z-score < −3SD at ART initiation^2^							0.01077
Yes	5.0(2.4–8.8)	7.2(4.0–11.6)	9.4(5.7–14.3)	16.6(11.4–22.6)	21.0(14.8–27.8)	NA	
No	2.3(1.5–3.5)	3.4(2.3–4.8)	5.7(4.2–7.3)	9.8(7.9–12.1)	13.2(10.9–15.8)	20.1(16.3–24.1)	
Regimen at Baseline^3^							< 0.0001
1NNRTI + 2NRTI’s	2.2(1.3–3.3)	3.2(2.2–4.5)	5.1(3.8–6.7)	8.6(6.9–10.6)	12.1(10.0–14.6)	19.0(15.3–23.0)	
LPV/R	1.8(0.1–8.7)	5.6(1.4–14.1)	11.6(4.6–22.1)	30.5(17.0–45.2)	34.0(19.4–49.2)	43.9(20.6–65.2)	
3NRTI	10.3(4.7–18.3)	12.9(6.5–21.4)	17.0(9.5–26.2)	27.5(18.0–37.9)	30.5(20.4–41.2)	N/A	
Year of ART Initiation^4^							< 0.0001
2003–2005	0	0	0	0	2.8(0.5–8.8)	12.7(6.1–21.6)	
2006–2009	2.9(1.7–4.7)	3.7(2.3–5.6)	5.9(4.1–8.2)	8.6(6.4–11.3)	12.2(9.5–15.2)	N/A	
2010–2012	2.6(1.2–4.9)	4.3(2.4–7.0)	6.2(3.9–9.4)	14.9(11.1–19.2)	19.0(14.3–24.1)	N/A	
2013–2015	4.0(1.6–8.0)	6.8(4.0–9.4)	11.5(6.8–17.4)	N/A	N/A	N/A	
Access to Phone^5^							< 0.0001
Yes	2.6(1.6–3.9)	3.3(2.1–4.9)	4.6(3.2–6.3)	8.2(6.3–10.5)	10.9(8.6–13.5)	17.5(13.4–22.1)	
No	3.3(1.7–5.6)	5.7(3.6–8.6)	10.1(7.1–13.7)	17.0(13.0–21.4)	22.4(17.7–27.3)	29.1(22.3–36.2)	
HIV Disclosed at Baseline^6^							0.0266
Yes	0.5(0.04–2.7)	0.5(0.04–2.7)	0.5(0.04–2.7)	3.8(1.7–7.4)	7.0(3.8–11.5)	18.5(11.3–27.0)	
No	3.3(2.3–4.7)	4.8(3.5–6.5)	7.7(6.0–9.6)	12.7(10.5–15.1)	16.4(13.8–19.2)	21.2(17.2–25.6)	

*Cumulative incidence estimates and confidence intervals derived from Fig. 2

Children who commenced ART in recent years (2013–2015) had higher cumulative incidence of LTFU [11.5%% (95% CI:6.8–17.4)] after 1 year, compared to those who begun treatment from 2010 to 2012 [6.2% (95% CI: 3.9–9.4)] and those who begun treatment from 2006 to 2009 [5.9% (95% CI: 4.1–8.2)] (p < 0.0001) during the same period of time. Lack of access to a phone resulted in higher cumulative incidence of 10.1% (95% CI: 7.1–13.7) compared to those whose caregivers owned phones [4.6% (95% CI: 3.2–6.3)], (p < 0.0001). Older children aged 7 years and above who did not know their HIV status had higher cumulative incidence of 7.7% (95% CI: 6.0–9.6) compared to those who knew their HIV status [0.5% (95% CI: 0.04–2.7) [16]], (*p* = 0.0266) (Fig. 2, Table 4).

After adjusting for age, baseline CD4, baseline hemoglobin, HIV disclosure status, nutritional status and WHO clinical stage, children who started treatment between 2013 and 2015, had the highest hazard ratio of loss LTFU, (aHR = 5.6; 95% CI:2.2–14.1), compared to those who started treatment between 2003 and 2005 (Table 5). Compared to children whose care givers owned phones, children without phones were 80% more likely to be LTFU (aHR = 1.8; 95% CI: 1.3–2.5). Among children above the age of 7, those who did not know their HIV status had 90% higher hazard of loss to follow-up in the univariable analysis when compared to their counterparts who knew their HIV status at baseline (HR = 1.9; 95% CI:1.2–2.9). However, this was not statistically significant in the multivariable analysis (Table 5).

**Table 5:** Factors Associated with Loss to Follow-up Among Children on ART at LCH (2003–2015)

Predictor	*N*	LTFU	Unadjusted HR (95% C1)	*p*-value	Adjusted HR (95% CI)	*p*-value
Gender (N%)						
Male	519	92	1.2 (0.9–1.7)	0.1929		
Female	520	75	Ref			
Age Continuous			0.94 (0.91–0.98)	**0.001**		
Categorical Age						
< 1 year	179	45	2.7 (1.8–3.9)	**<.0001**		
1–5 years	387	59	1. 3 (0.9–1.8)	0.1717		
> 5 years	473	59	Ref			
Year of ART Initiation						
2003–2005	71	10	Ref		Ref	
2006–2009	512	81	2.2 (1.0–4.5)	**0.0293**	2.3 (1.1–4.9)	0.0316
2010–2012	305	53	3.6 (1.7–7.7)	**0.0009**	3.5 (1.6–7.9)	**0.002**
2013–2016	151	20	5. 1 (2.2–11.9)	**0.0002**	5.6 (2.2–14.1)	**0.0002**
Low-weight < −3SD						
Yes	182	34	**1.6 (1.1–2.4)**	**0.0117**	1.1 (0.7–1.6)	0.7145
No	857	133	**Ref**		Ref	
Baseline CD4%						
< 15%	307	58	1.9 (1.1–3.4)	**0.03**		
15–25%	261	36	1.3 (0.7–2.4)	0.3675		
> =25%	112	14	Ref			
missing	359	59				
Baseline Hemoglobin (Continuous)	1039		0.87 (0.7–0.9)	**0.0036**	0.9 (0.8–1.0)	0.0694
Baseline Hemoglobin (Categorical)						
< =8	335	67	1.4 (1.03–1.9)	**0.0477**	1.2 (0.9–1.7)	0.1958
> 8	704	100	Ref			
WHO Stage at Baseline						
Stage 1 and 2	377	57	Ref			
Stage 3	471	70	0.9 (0.6–1.3)	0.5036		
Stage 4	177	36	1.6 (1.0–2.3)	0.0356		
TB at Baseline						
Yes	318	46	Ref			
No	721	118	0.8 (0.5–1.0)	0.1279		
Access to Phone						
Yes	705	85	Ref		Ref	
No	340	82	**2.1 (1.6–2.9)**	**<.0001**	**1.8 (1.3–2.5)**	**0.0002**
Regimen at Baseline						
1NNRTI + 2NRTi’s	907	124	Ref		Ref	
LPV/R Based Regimen	54	16	**2.9 (1.7–4.9)**	**<.0001**	**1.9 (1.0–3.3)**	**0.0374**
3NRTIs	78	25	**3.4 (2.3–5.4)**	**<.0001**	**2.6 (1.6–4.2)**	**0.0001**
Mother Took ART for PMTCT						
Yes	162	31	Ref			
No	877	136	0.6 (0.4–0.9)	**0.03**	1.0 (0.6–1.5)	
HIV Status Disclosed at Baseline^a^						
Yes	191	24	Ref		Ref	
No	854	143	1.9 (1.2–2.9)	**0.004**	1.4 (0.9–2.4)	0.1704

This Model was stratified by WHO stage and baseline age

^a^Only children aged above 7 years’ old

## Discussion

This study documents treatment outcomes during program expansion (2003–2015), in a routine pediatric HIV treatment setting in high HIV burden district in Zambia. At least 16% of children were LTFU, 7% died and 56% were still active on treatment. We document outcomes of children who were LTFU; 26% died, 47% were confirmed treatment defaulters, 13% continued ART at other clinics and 14% had continued treatment with gaps at other ART clinics. The cumulative incidence of LTFU increased with longer duration on ART. Initiation of ART between 2013 and 2015 and lack of phone ownership was associated with increased hazard of LTFU.

Loss to follow-up of more than 10% is regarded as substantially high based on the current adult and pediatric ART treatment guidelines [17]. Although we did not expect to find such a high LTFU in a well-organized pediatric HIV treatment program, our findings are consistent with findings in other Sub-Sahara settings [18]. The cumulative incidence of loss to follow-up increased as duration on ART increased. The highest hazard of LTFU was among children who started ART during recent years (2013 and 2015) [19]. This finding has important programmatic implications because the higher hazard of LTFU occurred in a period when the test and treat policy for children and pregnant women was fully operationalized. The number of children commencing treatment also declined during this period, but likely as a result of reduced infant infections due to successes in prevention of mother to child HIV transmission [16, 20].

In this period all children who were confirmed HIV positive were commenced on ART in contrast to previous years where ART eligibility was based on clinical evaluation and immunological staging [21, 22]. Prior to 2013 ART was only provided to very sick children whose caregivers saw their clinical improvement following ART and associated survival with the treatment, therefore were likely highly motivated to stay on treatment [23]. These findings are similar to those of a study done in South Africa in which children commenced on ART in recent years experienced poorer retention in care [20]. This could be because children commenced on ART in the test and treat era are likely healthier and their care givers may not be motivated to take them for medication pickups and reviews.

After tracing children who were LTFU, the proportion of children who were found to have died was substantially high (26%). A similar study from Malawi which retrospectively analyzed data for children enrolled over a duration of 4 years found that 11% of children LTFU had died. A meta-analysis of studies from different settings found that 21.8% of LTFU children had died 4 years after their last hospital visit [18]. The difference between the study from Malawi and our study is that our study had a longer duration of observation of 12 years as compared to 4 years in Malawi. Our study covers the outcomes of children who were on treatment before the test and treat era when ART eligibility was based on immunological criteria. Our findings show the importance of early tracing of LTFU children in treatment programs because mortality remains high, in this case, mortality among the LTFU was higher than among the children who were retained in care (26% vs 7% respectively).

The proportion of defaulters was high among the traced LTFU children (47%). This may indicate failure to engage in care. Engagement in care is critical during the early weeks and months after ART initiation. In a case control study from Botswana, it was found that 47.6% (*n* = 51) of the children who were LTFU failed to engage after just one clinic visit as compared to 1% (*n* = 2) in the control group who engaged in care [4]. The authors suggested that engagement can be improved by addressing personal concerns at the initial and follow-up clinical visits [4]. Other researchers proposed early tracing of patients who missed their appointments [6] and use of risk scores to identify patients at risk of LTFU at baseline and provide individualized risk assessment [24]. Risk scores would be useful in the PCOE clinic. In addition to risk scores, we suggest that dealing with stigma and child disclosure related issues early in the course of treatment and engagement in facility and community treatment support groups would improve engagement in care [25].

In our program, adherence counseling was performed by trained nurse counsellors at each visit. The pharmacists and clinicians also reinforced adherence by performing pill counts and asking specific adherence related questions and addressing any questions from the patients and caregivers. The clinic outreach team also reinforced adherence through a combination of home visits and phone calls aimed at engaging patients in care. In addition, the clinic carried out seminars and workshops and stays in touch with community empowerment opportunities and activities aimed at creating support networks for families of children living with HIV.

Although our finding that 13% of the children silently continued treatment was a threat to retention in care, this could also be addressed by improving engagement in care early and providing easily accessible channels of communication between caregivers and the health facility staff. A study done in South Africa suggested that with the scale-up of ART services, an increasing proportion of patients were transferring between ART services and there is need to strengthen tracking systems to ensure that patients planning transfer receive thorough clinical evaluation and are linked to appropriate services at their destination [26].

Access to a mobile phone improved retention in care in this population. Children whose caregivers did not own a phone had higher adjusted hazard ratio of loss to follow-up aHR = 1.8 (1.3–2.5). In Malawi, they found that access to a phone doubled success of tracing a lost patient [7]. This is supported by results of a meta-analysis of studies from Sub-Sahara in which 77% of the lost patients were successfully traced using a combination of phone calls and home visits [8] and in our study we successfully traced patients using a combination of phone calls and home visits. Based on these findings, routine clinical settings can improve patient retention by maintaining open communication with patients. While access to a phones makes contacting patients easier, we recognize that phones are not easily accessible to a lot of families in our setting. Our program has been able to communicate with caregivers who do not own phones through home visits, engaging them in community support groups and allowing them to bring their children to our clinic for other sick and well child visits. Therefore, as mobile phone coverage expands, we recommend that program managers plan for use of modern electronic technology in the follow-up of patients in addition to the current strategies.

In this sample only 18% of the children knew their HIV status at baseline and these children had better retention in care than those who did not know their HIV status aHR = 1.9 (95% CI:1.2–2.9). Disclosure of HIV infection status to a child is an incremental process that starts with partial disclosure to younger children leading to full disclosure for older children [27, 28]. In our study, only children who were at least 7 years old were reported to be fully disclosed. HIV disclosure rates to children in Sub-Sahara Africa remain low, some studies attribute this to health worker and caregiver lack of skills to disclose to children [29, 30]. Training health workers and caregivers in disclosure skills has improved disclosure rates in most places [29, 30].

A major strength of our study is that the data covers a long period of over 12-years in a routine clinical setting. Most studies in children were conducted over shorter periods of time [5, 6, 31]. The PCOE clinic had programs to trace children who missed appointments every week and up to 6 months after they missed their last scheduled visit. The competing risks approach allowed us to study the cumulative incidence of all the treatment outcomes at the same time (mortality, LTFU and transfer).

A major limitation of our study is that we did not report exact dates when the children were traced and therefore, we cannot estimate number of days from LTFU to tracing. As a result, we could not model the final tracing outcomes but modeled the initial outcomes in the competing risks models and the Cox regression. However, our findings are still adequate for us to make reasonable conclusions regarding risk factors of LTFU among children on ART in routine clinical settings.

## Conclusion

LTFU was higher than expected in an optimally functioning pediatric HIV treatment program. Among the children LTFU, mortality and default were substantially high. Children who started treatment in recent years (2013–2015) had the highest hazard of LTFU. Lack of access to a phone and non-disclosure of HIV-status to the index child was associated with higher hazards of LTFU over the study period. We recommend re-enforcement of client counselling and focused follow-up strategies using modern technology such as mobile phones as adjunct to current approaches.

## Data Availability

The data that support the findings of this study are available from Livingstone Central Hospital, but restrictions apply to the availability of these data, which were used under license for the current study and currently not publicly available. Data are however available from the authors upon reasonable request and with the permission of the Zambia National Research Authority.

## Abbreviations

AIDS: Acquired immunodeficiency syndrome
ART: Antiretroviral therapy
HIV: Human immunodeficiency virus
LCH: Livingstone Central Hospital
NNRTI: Non-nucleoside reverse transcriptase inhibitors
NRTI: Nucleoside reverse transcriptase inhibitors
PCOE: Pediatric Center of Excellence
PMTCT: Prevention of Mother to Child Transmission of HIV
WHO: World Health Organization

## References

[1] Abrams EJ, Strasser S (2015). 90–90-90--Charting a steady course to end the paediatric HIV epidemic. J Int AIDS Soc.

[2] Fox Matthew P., Rosen Sydney (2015). Systematic review of retention of pediatric patients on HIV treatment in low and middle-income countries 2008–2013. AIDS.

[3] Geng EH, Nash D, Kambugu A, Zhang Y, Braitstein P, Christopoulos KA (2010). Retention in Care Among HIV-Infected Patients in Resource-Limited Settings: Emerging Insights and New Directions. Current HIV/AIDS Rep.

[4] Machine Edwin Masese, Gillespie Susan L., Homedes Nuria, Selwyn Beatrice J., Ross Michael W., Anabwani Gabriel, Schutze Gordon, Kline Mark W. (2016). Lost to follow-up: failure to engage children in care in the first three months of diagnosis. AIDS Care.

[5] Braitstein P, Songok J, Vreeman R, Wools-Kaloustian K, Koskei P, Walusuna L (2011). ‘Wamepotea’ (They have become lost): Outcomes of HIV-positive and HIV-exposed children lost to follow-up from a large HIV treatment program in western Kenya. J Acquired Immune Defic Syndr.

[6] Ardura-Garcia C, Feldacker C, Tweya H, Chaweza T, Kalulu M, Phiri S (2015). Implementation and Operational Research: Early Tracing of Children Lost to Follow-Up From Antiretroviral Treatment: True Outcomes and Future Risks. J Acquired Immune Defic Syndr.

[7] Weigel R, Hochgesang M, Brinkhof MWG, Hosseinipour MC, Boxshall M, Mhango E (2011). Outcomes and associated risk factors of patients traced after being lost to follow-up from antiretroviral treatment in Lilongwe, Malawi. BMC Infect Dis.

[8] Zürcher K, Mooser A, Anderegg N, Tymejczyk O, Couvillon MJ, Nash D (2017). Outcomes of HIV-positive patients lost to follow-up in African treatment programmes. Trop Med Int Health.

[9] Kranzer K, Meghji J, Bandason T, Dauya E, Mungofa S, Busza J (2014). Barriers to provider-initiated testing and counselling for children in a high HIV prevalence setting: a mixed methods study. PLoS Med.

[10] Kankasa C, Carter RJ, Briggs N, Bulterys M, Chama E, Cooper ER (2009). Routine Offering of HIV Testing to Hospitalized Pediatric Patients at University Teaching Hospital, Lusaka, Zambia: Acceptability and Feasibility. J Acquired Immune Defic Syndr.

[11] Ministry of Health z. Zambian Guidelines for Antiretroviral Therapy of HIV Infection in Infants and Children:Towards Universal Access- Recommendations for a Public Health Approach 2007. 8/27/2017.

[12] Ministry of Health Z (2013). Zambia Consolidated Guidelines for Treatment and Prevention of HIV Infection.

[13] Andersen PK, Geskus RB, de Witte T, Putter H (2012). Competing risks in epidemiology: possibilities and pitfalls. Int J Epidemiol.

[14] Team RC (2017). R: a language and environment for statistical computing.

[15] Therneau Terry M. A Package for Survival Analysis in S_ 2015; version 2.38.https://CRAN.R-project.org/package=survival. Accessed 25 Aug 2017.

[16] Diallo K, Kim AA, Lecher S, Ellenberger D, Beard RS, Dale H, et al. Early Diagnosis of HIV Infection in Infants - One Caribbean and Six Sub-Saharan African Countries, 2011–2015. MMWR Morb Mortal Wkly Rep. 2016;65(46):1285–90 https://www.cdc.gov/mmwr/volumes/65/wr/mm6546a2.htm. 10.15585/mmwr.mm6546a2 27880749

[17] Joint United Nations Programme on HIV/AIDS U (2014). 90–90-90 An ambitious target to help end the AIDS epidemic.

[18] Chammartin F, Zürcher K, Keiser O, Weigel R, Chu K, Kiragga AN (2018). Outcomes of Patients Lost to Follow-up in African Antiretroviral Therapy Programs: Individual Patient Data Meta-analysis. Clin Infect Dis.

[19] W.H.O. Use of Antiretroviral Drugs for treating Pregnant Women and Preventing HIV infection in infants. Geneva: W.H.O; 2012. 5/2/2017

[20] Lilian RR, Mutasa B, Railton J, Mongwe W, McIntyre JA, Struthers HE, Peters RPH (2017). A 10-year cohort analysis of routine paediatric ART data in a rural South African setting. Epidemiol Infect.

[21] Bolton-Moore C, Mubiana-Mbewe M, Cantrell RA (2007). Clinical outcomes and cd4 cell response in children receiving antiretroviral therapy at primary health care facilities in zambia. JAMA.

[22] Stringer JA, Zulu I, Levy J (2006). Rapid scale-up of antiretroviral therapy at primary care sites in zambia: Feasibility and early outcomes. JAMA.

[23] Phiri N, Haas AD, Msukwa MT, Tenthani L, Keiser O, Tal K (2018). “I found that I was well and strong”: Women’s motivations for remaining on ART under Option B+ in Malawi. Plos One.

[24] McNairy ML, Abrams EJ, Rabkin M, El-Sadr WM (2017). Clinical decision tools are needed to identify HIV-positive patients at high risk for poor outcomes after initiation of antiretroviral therapy. Plos Med.

[25] Grimwood A, Fatti G, Mothibi E, Malahlela M, Shea J, Eley B (2012). Community adherence support improves programme retention in children on antiretroviral treatment: a multicentre cohort study in South Africa. J Int AIDS Soc.

[26] Nglazi MD, Kaplan R, Orrell C, Myer L, Wood R, Bekker L-G (2013). Increasing Transfers-Out from an Antiretroviral Treatment Service in South Africa: Patient Characteristics and Rates of Virological Non-Suppression. Plos One.

[27] Murnane PM, Sigamoney S-L, Pinillos F, Shiau S, Strehlau R, Patel F (2017). Extent of disclosure: what perinatally HIV-infected children have been told about their own HIV status. AIDS Care.

[28] Atwiine B, Kiwanuka J, Musinguzi N, Atwine D, Haberer JE (2015). Understanding the role of age in HIV disclosure rates and patterns for HIV-infected children in southwestern Uganda. AIDS Care.

[29] Madiba Sphiwe (2016). Caregivers Lack of Disclosure Skills Delays Disclosure to Children with Perinatal HIV in Resource-Limited Communities: Multicenter Qualitative Data from South Africa and Botswana. Nursing Research and Practice.

[30] Mweemba M, Musheke MM, Michelo C, Halwiindi H, Mweemba O, Zulu JM (2015). “When am I going to stop taking the drug?” Enablers, barriers and processes of disclosure of HIV status by caregivers to adolescents in a rural district in Zambia. BMC Public Health.

[31] Fenner L, Brinkhof MWG, Keiser O, Weigel R, Cornell M, Moultrie H (2010). Early mortality and loss to follow-up in HIV-infected children starting antiretroviral therapy in Southern Africa. J Acquired immune Defic Syndr.

